# Study protocol for Young & Strong: a cluster randomized design to increase attention to unique issues faced by young women with newly diagnosed breast cancer

**DOI:** 10.1186/s12889-015-1346-9

**Published:** 2015-01-31

**Authors:** Mary L Greaney, Kim Sprunck-Harrild, Kathryn J Ruddy, Jennifer Ligibel, William T Barry, Emily Baker, Meghan Meyer, Karen M Emmons, Ann H Partridge

**Affiliations:** University of Rhode Island, Kingston, RI 02881 USA; Dana-Farber Cancer Institute, Boston, MA 02215 USA; Mayo Clinic, Rochester, MN USA; Harvard School of Public Health, Boston, MA USA; Harvard Medical School, Boston, MA USA

**Keywords:** Breast cancer, Oncology, Young women, Fertility, Exercise, Psychosocial distress, Patient communication

## Abstract

**Background:**

Each year, approximately 11% of women diagnosed with breast cancer in the United States are 45 years of age or younger. These women have concerns specific to or accentuated by their age, including fertility-related concerns, and have higher rates of psychosocial distress than women diagnosed at older ages. Current guidelines recommend that fertility risks be considered early in all treatment plans; however, the extant research indicates that attention to fertility by the healthcare team is limited. Importantly, attention to fertility may be a proxy for whether or not other important issues warranting attention in younger women with breast cancer are addressed, including genetic risks, psychosocial distress, sexual functioning, and body image concerns. The Young & Strong study tests the efficacy of an intervention designed for young women recently diagnosed with breast cancer and their oncologists with the intention to: 1) increase attention to fertility as an important surrogate for other issues facing young women, 2) educate and support young women and their providers, and 3) reduce psychosocial distress among young women with breast cancer.

**Methods/Design:**

The study employs a cluster randomized design including 14 academic institutions and 40 community sites across the U.S. assigned to either the study intervention arm or contact-time comparison intervention arm. Academic institutions enroll up to 15 patients per site while community sites enroll up to 10 patients. Patient eligibility requirements include: an initial diagnosis of stage I-III invasive breast cancer within three months prior, without a known recurrence or metastatic breast cancer; 18–45 years of age at diagnosis; ability to read and write in English. The primary outcome is oncologists’ attention to fertility concerns as determined by medical record review. Secondary outcomes include differences in patient satisfaction with care and psychosocial distress between the two study arms.

**Discussion:**

Study findings will provide valuable insight into how to increase attention to fertility and other issues specific to young women with breast cancer and how to improve doctor-patient communication around these issues, which may promote better quality of care for this population.

**Trial registration:**

NCT01647607. Registered July 19, 2012.

## Background

An estimated 232,340 women living in the in the United States were diagnosed with breast cancer in 2013 [1], approximately 11% of whom were under the age of 45 [2]. Young women with breast cancer have more aggressive disease and lower rates of survival than older women with breast cancer [3]. As a result, this population usually receives more therapy than women who are diagnosed when older (aged 46+) [4,5]. These treatments are physically and emotionally taxing, particularly given the lower survival rates for young women. Additionally, young women with breast cancer face problems unique to or accentuated by their young age, such as completing their education, developing a career, and parenting young children [6]. They are often concerned that their health care providers have not received adequate training on health issues specific to their young age, as the median age at breast cancer diagnosis is 61 [7]. Some young women may also place high importance on body image and attractiveness, which can be detrimentally impacted by cancer treatments. Additionally, younger women can feel isolated from other breast cancer patients due to their relative youth [8]. These life factors and stressors contribute to psychosocial distress, which is greater in this population than in older women with breast cancer at both diagnosis and follow-up [9-13]. Due to these multiple unique issues, the Centers for Disease Control and Prevention has stated that survivorship issues in young breast cancer patients are an important focus for further research and intervention [14].

In addition to the specific concerns outlined above, young women diagnosed with breast cancer are of childbearing age and may desire biologic children. Cancer treatments can eliminate or delay fertility in premenopausal women [15,16]. Thus, fertility-related concerns must be addressed early to ensure that opportunities for fertility preservation are not missed [17,18]. Although the current recommendation is that fertility risks and concerns be considered in all treatment plans [17], in practice, risks to fertility and fertility options may not be discussed or, if addressed, may be addressed inadequately [13,19-21]. There are a number of factors that may impair attention to fertility: providers may be uncomfortable discussing loss of fertility or may simply forget that this could be an important topic for the patient, given her life stage; providers may also lack knowledge about this topic or feel like they do not have the time to adequately assist patients in making fertility-related decisions, including decisions related to fertility preservation methods [22,23].

As well as its direct impact on fertility, attention to fertility is important because it may be a proxy for whether or not other issues relevant to younger women with breast cancer are being addressed, including genetic risks, psychosocial distress, and sexual functioning. There has been increased interest to uniformly include personalized psychosocial support, counseling on genetic risks, and attention to fertility in treatment plans of young women with breast cancer [24]. Nonetheless, recent Quality Oncology Practice Initiative Network audit data determined that documentation of attention to fertility was less than 30% among the majority of audited practices, and the rate was 0% in more than 50% of the practices (personal communication between ASCO staff and Dr. Partridge). High quality care for young women with breast cancer includes discussing these issues in a systematic and consistent manner, both at diagnosis and in follow-up. Increased attention to this complement of issues may lead to increased satisfaction with care and treatment decisions resulting in decreased distress and better overall quality of life for young women with breast cancer.

Based on our prior experience with designing a unique in-clinic program for young women with breast cancer [25,26], we developed Young & Strong, a print-based educational and supportive care intervention with a corresponding website for young women (18–45 years of age) newly diagnosed breast cancer and their providers. The intervention addresses issues salient to this population (e.g., body image, child care, fertility, education, and career issues). We hypothesize that an intervention that simultaneously increases providers’ awareness of salient issues and educates and encourages patients to communicate with their doctors about these important topics has the potential to reduce psychosocial distress and improve satisfaction with care among young breast cancer patients. Therefore, the primary aim of the Young & Strong study is to evaluate the effect of the Young Women’s Intervention (YWI) on oncologists’ attention to fertility issues compared to a contact-time comparison intervention focusing on physical activity (PAI). Secondary aims include exploring how YWI affects attention to other issues important to young women with breast cancer, including genetic testing, satisfaction with care, distress, and quality of life.

## Methods

### Study design

**Figure 1:**
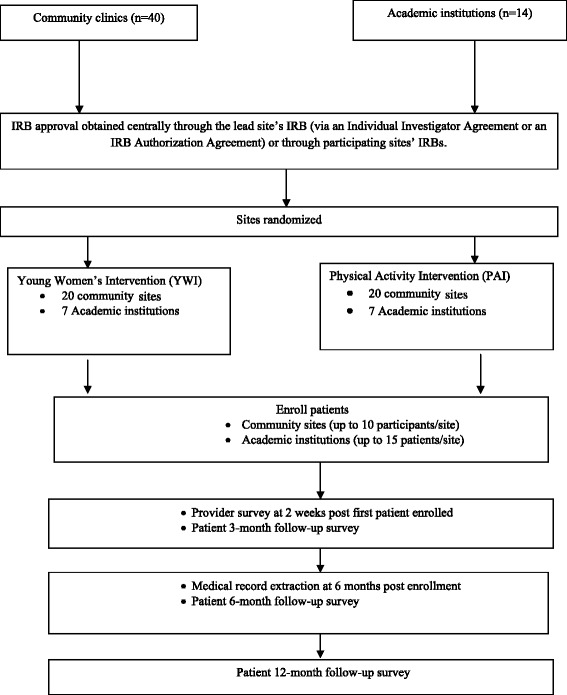
**Flow of the young & strong study.**

The Young & Strong study is a multi-site, two arm, cluster randomized clinical trial with the practice site being the unit of randomization (see Figure 1). The study compares the YWI arm to a contact-time, physical activity intervention (PAI) arm. Employing a contact-time comparison intervention ensures that study results are not a function of time and attention given to patients due to their involvement in the study.

### Identification of sites

**Figure 2:**
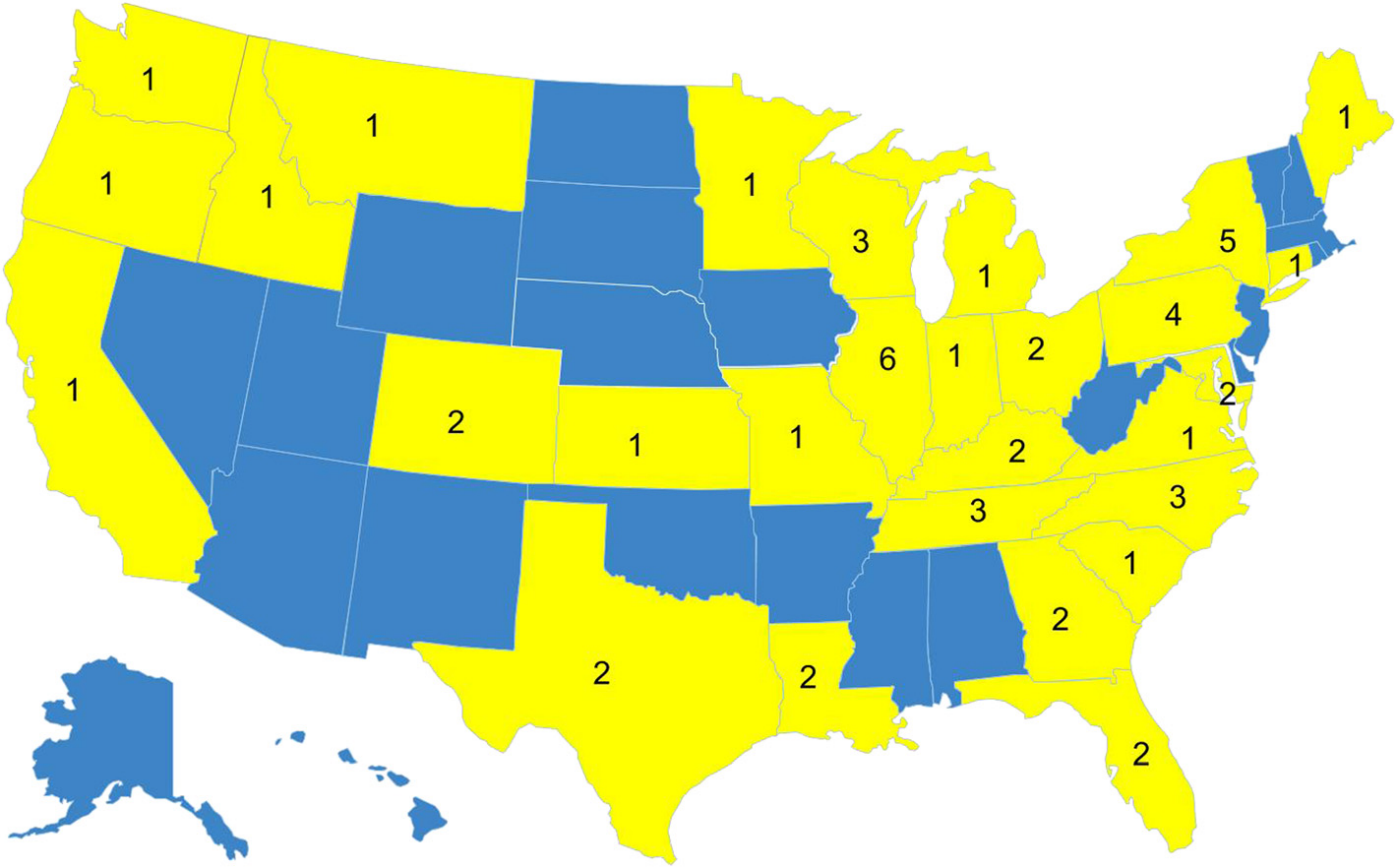
**Location of sites participating in the Young & Strong study.**

To reach a variety of patients, we are implementing the study in both academic institutions (n = 14) and community sites (n = 40) across the United States (see Figure 2).

Sites were identified in one of three ways. First, key contacts from national cancer organizations (i.e., National Cancer Institute Community Cancer Center Program, American Society of Clinical Oncology (ASCO) Quality Oncology Practice Initiative Network, and Alliance Cancer Control Program) sent introductory emails to member sites describing the study and inviting interested sites to contact the principal investigator (PI). Second, the PI directly contacted academic institutions and community sites across the country serving economically and racially diverse populations. Third, representatives from interested sites who learned of the study contacted the study PI directly.

### Site participation requirements

Sites have a local PI who ensures that the site adheres to all study protocols and obtains Institutional Review Board (IRB) approval. The study is approved by the Dana-Farber Cancer Institute IRB, which oversees a majority of study sites via Institutional Authorization Agreements (or Individual Investigator Agreements); some sites maintain their own institutional review. Additionally, sites: a) agree to be randomized to one of the two intervention arms; b) identify a site coordinator to manage the study locally; c) approach eligible patients until recruitment goals are met or the study stops recruitment, whichever comes first; d) obtain informed consent from interested patients and administer the baseline survey on site; e) distribute intervention materials; f) assist in follow-up of study participants as needed; g) transmit medical records of study participants to the lead site for review; h) facilitate medical oncology provider interaction with the intervention and study including alerting them of a patient’s enrollment and encouraging them to complete a one-time survey regarding the intervention.

### Lead site and participating site responsibilities

#### Lead site

The lead study site, a comprehensive cancer center located in the Northeastern part of the United States, is responsible for setting up all subcontracts and for training key staff at each participating site by telephone. After completion of this training, sites begin recruiting participants. Staff at the lead site conduct regular check-in calls during recruitment and beyond as needed and ensure that sites receive needed intervention materials.

#### Participating sites

Site coordinators identify eligible patients by reviewing new patient lists or through other usual recruitment procedures. Patients are eligible to participate if they are: a) within three months of initial diagnosis of stage I-III invasive breast cancer, without a known recurrence or metastatic breast cancer; b) 18–45 years of age at time of diagnosis; c) able to read and write in English. Additionally, patients must have their first apppointment with an oncologist particpating in the study after the site has completed the telephone training.

At each site, the site coordinator or other staff (e.g., research nurses) approaches eligible patients to discuss and explain study requirements. They obtain signed informed consent and then distribute the self-administered baseline survey to consented patients. Once the baseline survey is complete, the patient receives the intervention materials (described below). A copy of the enrollment materials, including consent form and baseline survey, are transmitted to the lead site where study staff officially registers the patient with the lead site’s registering board.

### Overview of the interventions

There is considerable work conducted across a range of intervention topics demonstrating that patient-oriented interventions, without parallel provider or systems-level interventions, are neither well-implemented nor sustained [27-29]. Thus, we designed a multi-pronged intervention, with both provider and patient intervention components.

The YWI focuses on issues important to young women newly diagnosed with breast cancer, including fertility and early menopause, pregnancy after breast cancer, psychosocial concerns (e.g., coping with anxiety, dating, career and education issues, and dealing with young children), genetic issues, body image, and sexual functioning. The physical activity intervention (PAI), developed for this study, is a contact-time comparison intervention designed to promote physical activity. Physical activity was selected as the focus of the contact-time comparison intervention due to the known benefits of physical activity for people with cancer, not unique to young women, including: improved cardiovascular fitness, quality of life, and self-esteem [30]; reduced depression, anxiety, and tiredness; and potential lower risk of recurrence and longer survival [31].

### Intervention development

To inform the design of intervention materials, we conducted four focus groups with young breast cancer survivors in Boston, Massachusetts (n = 36) [32] and key informant interviews (n = 20) with a racially, ethnically, and geographically diverse sample of young women with a history of breast cancer [33]. In addition, throughout intervention development, young breast cancer survivors and patient advocates reviewed intervention materials to ensure that all salient concerns were addressed. Once the intervention materials were developed, they were piloted at the lead site (MA) and three community sites (ME, TX, MA) to identify potential implementation issues, including procedural flow.

### Intervention components

#### Patient materials

**Figure 3:**
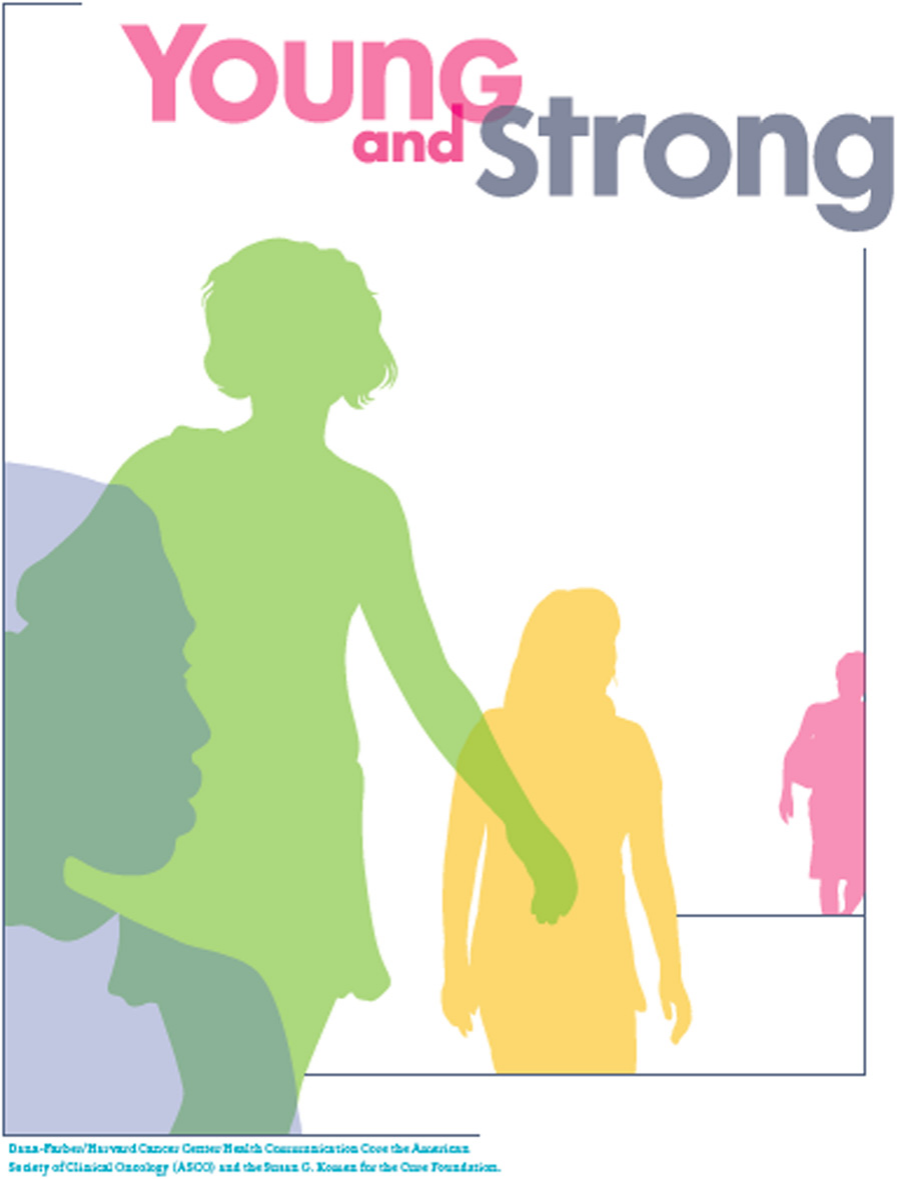
**Cover of the booklet for the young women’s intervention (YWI).**

**Figure 4:**
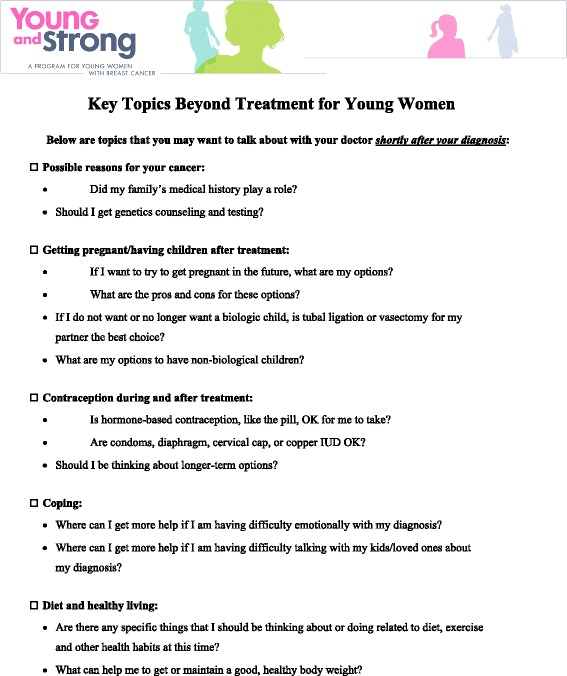
**Baseline checklist for patients in the young women’s intervention (YWI).**

**Figure 5:**
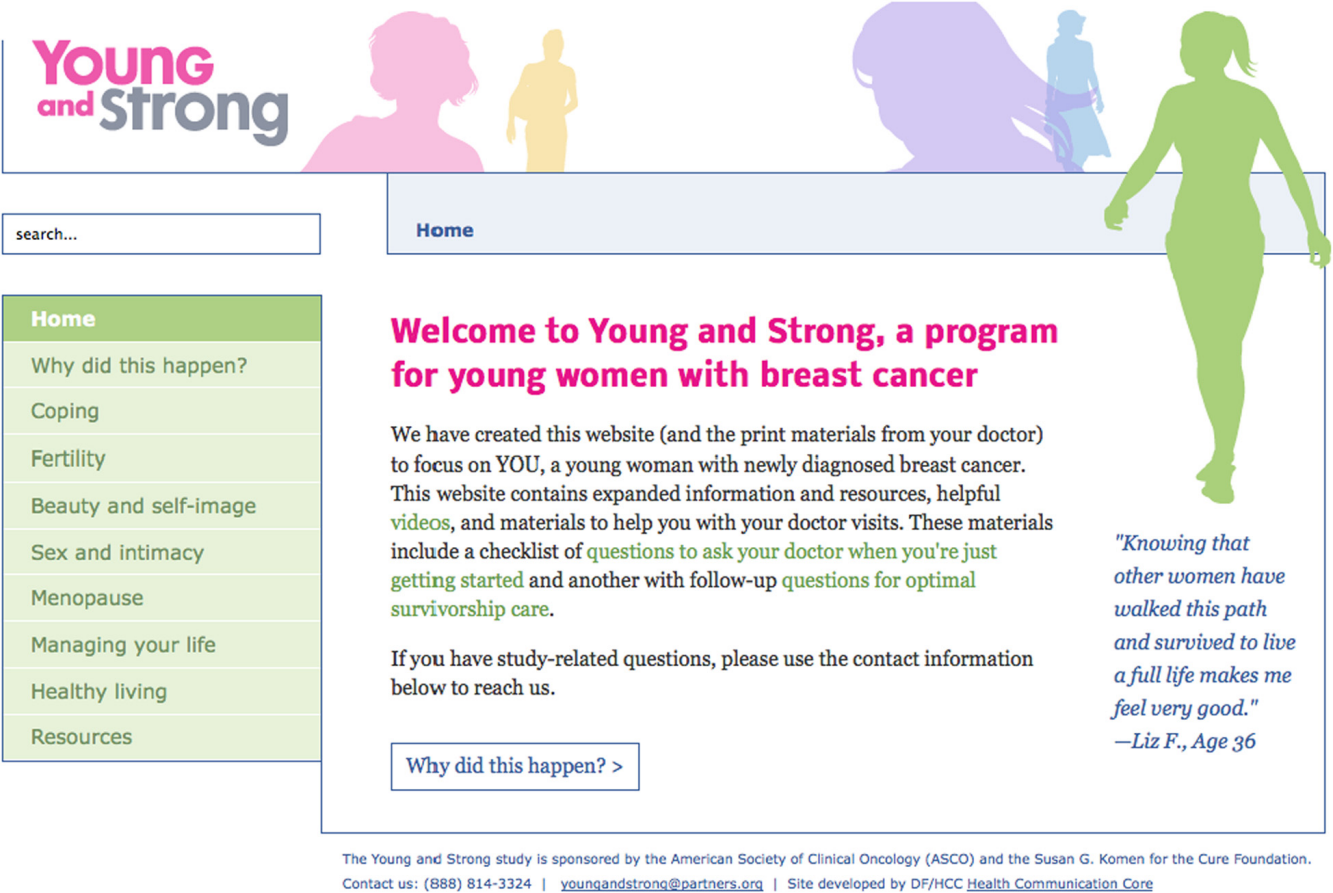
**Screen shot from the young women’s intervention (YWI) website.**

All intervention materials include key information designed to provide education and increase participants’ self-efficacy to discuss fertility/physical activity (arm specific) with their oncologists. The content of the intervention materials are specific to each arm (YWI vs. PAI) but include similar components: 1) a print booklet, the primary intervention component, 2) baseline/follow-up doctor visit checklists to encourage patients to discuss fertility/physical activity with their oncologists, and 3) a study-specific website that encompasses all of the information in the booklet plus additional information and resources, such as videos and downloadable PDFs (See Figures 3, 4 and 5). Materials are designed to be sustainable and disseminable. While the booklet, checklists, and website are specific to the intervention (YWI or PAI), and therefore hold distinct information, both sets of materials include information about the importance of social support and give pointed information about general healthy living topics, such as diet and nutrition and alcohol consumption. Participants receive intervention materials and login information for the website after consenting and completing the baseline assessment.

#### Oncologist materials

Intervention materials developed for oncologists include an introductory letter outlining the goals of the study, procedures, and their role; the arm-specific patient materials; clinical checklists which parallel the patient checklists so that topics can be discussed at baseline and follow-up appointments; and access to the website, with a special tab only accessible to clinicians. This tab holds videos by leading researchers in the field discussing the importance of addressing fertility or physical activity (dependent on study arm), additional oncologist- specific materials such as medical text related to the specific topic areas, and downloadable PDFs of the checklists. PDFs of the checklists. The study also provided email access to medical experts for consultation in young women’s issues and exercise among breast cancer patients.

### Statistical design

The number of sites (14 academic institutions and 40 community sites) and the sample size at each site (up to 15 participants per academic institution, up to 10 participants per community site) were chosen to detect differences in attention to fertility at three months. We based our expected detection difference on the 2009 data from the Quality Oncology Practice Initiative Network. These data, from 156 practices, showed that only 30% of records reflected documentation of discussion about infertility risks prior to treatment with patients of reproductive age and nearly half of practices had a 0% rate of documentation of discussion of fertility risks or concerns. Compliance with this quality of care measure, discussion of fertility, has not substantially improved since the Spring 2009 evaluation (personal communication between ASCO staff and Dr. Partridge). Therefore, we estimate that among study participants who are seen at sites assigned to PAI, the attention to fertility rate at three months will be 10% and that the rate among sites randomized the YWI arm will be at least 40%. The sample size was selected to give adequate power to detect a difference of around 30% in attention to fertility at three months. As there are no data on within-practice correlation and practice size, we assumed a practice size of at least 15 within large academic sites and at least five within small community sites and a within-practice correlation of 0.50 to estimate power. With seven practices per arm from large academic sites and 20 practices per arm from small community sites (total of a minimum of 410 patients accrued at 54 sites), there is 80% power to detect a difference of 28%. Using calculations proposed by Donner and Buck [34], we have 90% power to detect the same difference if the within-practice correlation is 0.30.

### Measures

#### Attention to fertility as reported in medical records

The primary study outcome, attention to fertility within three months of their enrollment into the study will be assessed by medical record review. Medical record extractors, who will be blind to intervention arm, will determine whether the medical records include agreed upon notations indicating that fertility was addressed and will note the timing of these discussions with respect to diagnosis and treatment. Using a pre-populated list, they will note if fertility issues did not need to be discussed (i.e., patient had a bilateral oophorectomy prior to diagnosis). Reporting will be done in aggregate. We will also record additional items, including documentation of referral to genetic testing, discussion of contraception use, and attention to weight and exercise behaviors.

#### Participant surveys

Participants complete an initial baseline assessment and additional assessments at three, six, and twelve months post-enrollment. As an incentive, study participants receive a $10, $15, and $20 gift card for each follow-up assessment, respectively. To reduce participant burden, not all measures (see below) are assessed at each time point (Table 1). Patients primarily complete the follow-up surveys via a HIPAA compliant website or by telephone, with participants choosing their preferred modality. Patients are also able to complete follow-up surveys at clinic appointments if more convenient. Or, if participants express a preference to return completed surveys via mail, they may do so. Providers complete one five-minute survey, approximately two weeks after his/her first patient enrolls on the study. Each participating site receives $200 for each enrolled study participant to help cover administrative costs.

**Table 1:** **Measures and time of assessment in the young & strong study**

**Measure**	**Assessed by**	**Time of assessment**
Attention to fertility		
	• Medical Record Review	3- and 6-mo
	• Fertility Issues Survey [35]	3-, 6- and 12-mo
Anxiety, depression, and distress		
	• Hospital Anxiety and Depression Scale (HADS) [37]	BL, 3- and 12-mo
	• Center for Epidemiologic Studies Depression Scale (CES-D) [39]	BL, 3- and 12-mo
	• Perceived Stress Scale (PSS) [40]	BL, 3- and 12-mo
Health-related quality of life		
	• Cancer Rehabilitation Evaluation System-SF (CARES-SF) [41,42]	3- and 12-mo
Contraception		
	• Items used in prior work assessing methods of contraceptive use among young women with breast cancer at diagnosis and over time [36].	3- and 12-mo
Satisfaction with care		
	• Short-Form Patient Satisfaction Questionnaire (PSQ-18) [43]	3-mo
Physical activity		
	• Godin Leisure Time Physical Activity Questionnaire [44,45]	BL, 3-, 6- and 12-mo
	• Nurse’s Health Study Physical Activity Question Index [46]	BL, 12-mo
Intervention engagement		
	• Items used in prior work to gauge use of intervention materials [49]	3-mo
Socio-demographics		
	• United States Department of Health and Human Services Breast Cancer Core Questionnaire to assess age ethnicity, education, marital status, employment status [47,48]	BL
	• Participants’ perceived financial status of their household	
	• Ashkenazi ancestry	

Note: BL = baseline.

#### Attention to fertility as reported by patients

On all follow-up surveys, study participants complete the Fertility Issues Survey that was developed for premenopausal women undergoing treatment for early stage breast cancer, and assesses concern about fertility, impact of fertility issues on treatment decision-making, perceived risk of infertility following treatment, fertility history, and desire for future pregnancies [35]. Current contraception use is being assessed using a measure designed for an ongoing cohort study of young women with breast cancer [36].

#### Self-reported anxiety, depression and distress

Participants’ level of anxiety and depression are measured by the Hospital Anxiety and Depression Scale (HADS) [37], which is both reliable and valid [38]. A score of 11 or greater indicates a possible need for intervention. In addition, participants’ level of depressive symptomology in the preceding week will be assessed by the Center for Epidemiologic Studies Depression Scale (CES-D) [39]. The CES-D provides a summary score for level of depressive symptoms, with score ranges from 0–60. Scores > 16 indicate potentially clinically significant levels of depression. Participants also complete the Perceived Stress Scale (PSS) to assess the perceived level of stress in their lives in the preceding month. The PSS was designed to determine how unpredictable, uncontrollable, and overloaded respondents find their lives and also measure their current levels of experienced stress [40]. We are using the HADS scale because it is a valid and reliable measure of anxiety that we have used extensively in other studies with young women with breast cancer. We are using both the CES-D and PSS because these scales are more sensitive than the HADS to potential changes in depression and stress, respectively. While all measures (HADS, CES-D, and PSS) will be used in the data analysis, only the HADS will be used to immediately assess depressive symptoms in participants in order to notify their physician, as needed.

#### Health-related quality of life

Patients complete a modified version of the 59-item Cancer Rehabilitation Evaluation System-Short Form (CARES-SF) to evaluate health-related quality of life and concerns more specific to cancer survivorship [41,42]. Additionally, participants complete three scales from the long CARES: sexual interest, sexual dysfunction, and body image.

#### Satisfaction with care

Participants complete the Short-Form Patient Satisfaction Questionnaire (PSQ-18), an 18-item scale with seven subscales, including general satisfaction, technical quality, interpersonal manner, communication, financial aspects, time spent with doctor, and accessibility and convenience [43]. Additionally, participants report on provider attention to emotional health.

#### Physical activity

Patients complete the Godin Leisure-Time Physical Activity Questionnaire [44,45], which has been modified to include the average duration of activities at baseline and three, six, and 12-months post-enrollment. This questionnaire will be used to estimate weekly minutes of moderate and vigorous activity physical activity, with time spent in moderate physical activity being the primary outcome. Weekly minutes of vigorous activity and average weekly frequency of mild, moderate, and strenuous intensity exercise will also be examined to evaluate change in physical activity behaviors. Additionally, at baseline and 12-months post-enrollment, participants will complete the Nurse’s Health Study Physical Activity Question Index [46], which asks about the average time a person spent doing specific recreational activities in a typical week during the past year.

#### Socio-demographics characteristics

As part of the baseline survey, participants complete items in the Sociocultural Module of the United States Department of Health and Human Services Breast Cancer Core Questionnaire to assess age, ethnicity, education level, marital status, employment status, and income [47,48]. A participant’s perceived financial status of her household also was assessed, as was presence of Ashkenazi ancestry.

#### Intervention engagement

At three months post-enrollment, participants report the time they spent with the intervention materials, their engagement with the materials, their attention to the intervention messages, and the salience of the messages for them [49]. Salience will be measured by rating participants’ degree of interest in the intervention messages and how personally relevant the messages were. While the booklet is the primary intervention component, we will collect aggregate data on website usage. These data will provide valuable insight into the use of intervention components that will inform future interventions and research.

### Planned analyses

For the primary outcome, attention to fertility, the YWI and PAI will be compared in terms of attention to fertility issues as reported in the medical records using general estimating equations (GEE) to evaluate binomial proportions with clustered binary data [50]. GEEs will include terms for treatment arm and stratum, and population-averaged contrasts will be used to test the effects of the YWI versus PAI on attention to fertility. Generalized linear mixed models (GLMMs) will be used in subsequent analyses to describe the effect of the YWI with practice incorporated as a random effect, accounting for covariates such as patient demographics and, if appropriate, including variables in the secondary analyses as well as practice-level characteristics such as setting (urban, suburban, rural) and size of practice. Similar analyses will be conducted using attention to fertility as reported by patients. All analyses will be based on the intent-to-treat (ITT) population, consisting of all subjects at all randomized sites.

For the secondary analyses, examining the effect of the YWI on patients’ emotional health, distress, and quality of life, we will estimate proportions within each arm for each measure and report 95% confidence intervals. Lastly, to examine the effects of the PAI on improvement and maintenance of exercise behaviors, we will determine the mean change in exercise time at three months (post – pre) using a 2-sample t-test adjusting for within-practice correlation for both the PAI and YWI. We will examine change in exercise behaviors at six and twelve months to evaluate maintenance.

## Discussion

The Young & Strong study is designed to address the well-documented lack of attention to an important issue, fertility concerns, among a unique and relatively uncommon cancer population, young women with breast cancer. This study has been carefully designed to evaluate the role of a novel intervention, YWI, to improve targeted outcomes in young women with breast cancer and for dissemination in the future if it is successful. The use of a salient contact-time control intervention allows us to offer educational materials to providers and patients to engage them in the control content while at the same time not likely interfering with the main outcome of the intervention, attention to fertility. The use of a cluster randomized controlled trial, randomized by site, minimizes contamination between the intervention arm and the control arm that could result from provider- and site-level knowledge of the educational materials in both arms. We chose to develop an intervention for both patients and providers as we believe it is critical for both groups to engage in these discussions early in the course of treatment decision-making. The intervention is designed to be sustainable and disseminable; thus, if effective, we will work with an expanded network of provider organizations to disseminate the intervention to providers and patients. We believe that this is the first study to look at the rates of discussion of these topics between patients and providers while simultaneously intending to increase the discussion of these topics. This study is also further distinguished by the expansive reach into a variety of clinics across the country.

We believe that study findings will provide valuable insight into how to increase attention to fertility and other issues specific to young women with breast cancer and how to improve doctor-patient communication around these issues, which may promote better quality of care for this population. This work will add to the literature about how to improve care for young women with breast cancer, as well as serve as a novel model to improve care and overcome barriers to delivering optimal care for other unique groups of patients.

## Abbreviations

ASCO: American Society of Clinical Oncology
CES-D: Center for epidemiologic studies depression scale
HADS: Hospital anxiety and depression scale
IRB: Institutional review board
PAI: Physical activity intervention (the contact comparison intervention)
PI: Principal investigator
PSS: Perceived stress scale
PSQ-18: Short-form patient satisfaction questionnaire
QOPI: Quality Oncology Practice Initiative
YWI: Young women’s intervention
